# Genotyping of *Pseudomonas Aeruginosa* Strains As A Multidrug Resistant (MDR) Bacterium And Evaluating The Prevalence of Esbls and Some Virulence Factors Encoding Genes By PFGE and ERIC-PCR Methods

**DOI:** 10.22037/ijpr.2019.1100762

**Published:** 2019

**Authors:** Alireza Mokhtari, Kumarss Amini

**Affiliations:** a *Department of Microbiology,* *Faculty of Specialized Veterinary Science, Islamic Azad University, Science and Research Branch, Tehran, Iran.*; b *Department of Microbiology, School of Basic Sciences, Saveh Branch, Islamic Azad University, Saveh, Iran. *

**Keywords:** Pseudomonas aeruginosa, Multiplex-PCR, ESBLs, PFGE, ERIC-PCR

## Abstract

*Pseudomonas aeruginosa* is an important multi-drug resistant (MDR) opportunistic bacterium. 102 strains of *Pseudomonas aeruginosa* equally isolated from human and cow milk were subjected to Multiplex-PCR for detection of ESBLs and exoenzymes of U, T, S, OprI, and OprL, Integrons class A encoding genes and genotyping by the ERIC-PCR and PFGE methods. The disc diffusion and E-test based on CLSI (Clinical and Laboratory Standards Institute) were performed to identify the antibiotics’ resistant strains. Exotoxin A encoding gene was detected in more than 90% of the studied strains, exoenzyme S prevalence in isolated samples from animal (cow milk) was negative and the frequency of Exo Y, Exo T, and Exo U were 25%, 68.6%, and 68.6%, respectively. The frequency of VEB and GES encoding genes in human strains were detected as 3.9% and 0 by Multiplex-PCR, respectively. The highest resistance was seen to Ampicillin and Cefepime (100%) while the lowest was observed to Amikacin (80.3%). E-Test results on human and animal strains showed complete resistance to Meropenem and Ampicillin, respectively. Dendrogram of ERIC-PCR method on human isolated samples revealed 22 different groups. Frequency of Integron I encoding gene was detected as 21.5% and 1.96% in human and animal strains, respectively. In general, the present study showed the high value of genetic diversity among isolates from animal and human samples with different progenitors, but the clones classified in one cluster revealed the same source of infection.

## Introduction

Bacteria classified in genus *Pseudomonas* are aerobic Gram-negative, motile Gammaproteobacteria belonging to the family of *pseudomonaceae* which consists of over 191 species. Because of their great metabolic diversity, they can be found in every known niches as distilled water with the least nutritional contents. Within this genus, opportunistic *Pseudomonas aeruginosa* (*P*. *aeruginosa)* species which is considered as commensal bacteria in the skin and the lumen of digestive system, is one of the most known multidrug resistant (MDR) pathogens that causes different diseases in human, plants, and animals. In addition to the main causative agents of dairy cow mastitis, *P*. *aeruginosa* plays an important role in inducing acute and chronic mastitis in husbandries that causes huge economical loses worldwide. So far many research studies have been carried out to detect and eradicate *Pseudomonas* infection (1).

The ability of *P*. *aeruginosa* to survive and transmit between human hosts is the main cause of its huge prevalence of infection in hospitals and clinics (2). Genes encoding several enzymes that cause resistance to Beta-lactame antibiotics are classified in Class A such as VEB and GES enzymes encoding genes. Extended spectrum Beta-lactamases (ESBL) are enzymes produced by Gram-negative bacteria that are coded by plasmid gene sequences (3). VEB (Vietnam Extended Spectrum B-Lactamase) are coded by Integron I cassette (4) first detected in *Enterobacteriaceae* and *P*. *aeruginosa* from South Asia and then spread to North France (5). Another group of ESBLs are GES B-lactamases (Guyana Extended Spectrum B-Lactamase) which are specifically detected from *P*. *aeruginosa *strains and are not wide spread (6). Mutations in bacterial genome, enzymatic effects on the antibiotics to decrease their mode of actions, efflux pump mechanism for ejecting the antibiotic’s components, metabolites from bacterial cell changes in the target site of antibiotics, and transformation of motile genetic elements are the main factors for MDR pathogens enabling them to survive (7). Obtaining genes encoding factors interfering resistance against antibiotics occurs through mechanisms of transforming motile genetic elements such as plasmids, transposons, and integrons between bacteria in one bacterial population (8, 9, 7). Super- integrons are very old structures in bacterial genome that are found in a wide range of bacteria. Researchers believe that motile integrons are derived from super-integrons (10, 11). Integrons are motile genetic elements which transfer important sequences between bacteria which lead to MDR pathogen formation in Gram-negative species (12). Horizontal transfer of resistance encoding genes with Integrons, causes an increase in *P*. *aeruginosa* resistant strains, class I Integrons are the most common Integrons found in clinically isolated *P*. *aeroginosa* strains (9). Precise typing of *P*. *aeruginosa *different strains is needed for control and epidemiological monitoring of detecting new strains in a known bacterial population. Among different molecular-based methods for this purpose, ERIC PCR is one of the valuable rapid typing methods that can be used easily in every molecular biological equipped labors (13). PFGE (Pulse-Field Gel Electrophoresis) is a useful technique to separate large DNA molecules using periodically changing direction applying on an agarose gel that is considered as a golden standard test applying for subtyping different bacterial species such as *P. aerugnosa* strains (14). The mechanisms in which *P. aeruginosa* hide from the host’s immunologic responses is based on its ability to produce and excrete virulence factors such as exotoxin A and exoenzyme S and Alginate. Exotoxin A encoding gene is detected in more than 90% of known *P*. *aeroginosa* strains, coding the main venom of the bacteria, which causes a high rate of mortality in infected patients. Alginate as an exopolysaccharide synthesized by *P. aeruginosa* plays an important role in development, maintenance, and spread of *P. aeruginosa* biofilms, encoded by a group of related genes including algD. OprL and OprI are outer bacterial membrane lipoprotein I and peptidoglycan-associated lipoprotein (OprL) that can be used as biomarkers for detecting *P. aeruginosa* in a bacterial isolated population. 

The aim of this study was to genotype *Pseudomonas aeruginosa* strains isolated from human and animals, using PFGE on the Integrons class A positive samples and ERIC-PCR to compare the probable similarities of common progenitor sources. Identification of Beta-lactamases encoding genes of VEB and GES by multiplex-PCR and determination of antibiotic susceptibility of *Pseudomonas aeruginosa *strains using Disc Diffusion and E-Test were also carried out for detection of the MDR strains in order to rapidly prevent early infections. Also evaluation of virulence encoding gene factors frequency including exotoxin A, exoenzyme S, Alginate, OprL, and OprI were detected in isolated strains taken from patients and raw milk collected samples.

## Experimental

Sampling was done from patients all referred to major hospitals in Tehran, Iran. All samples were taken from urine, blood, lesion, and bronchial excretions and sent to a microbiological laboratory. The Samples were also taken from cow raw milk gathered from dairy cow husbandries around Tehran and Shahr-e-Kord city in west of Iran. First, all samples were cultured on Blood Agar (Merck, Germany), MacConkey Agar (Merck, Germany), and Cetrimid Agar (Merck, Germany) and were incubated for 24 h in 37 degree centigrade. Grown colonies were studied morphologically and after the Gram staining method, the Gram and lactose negative colonies were selected and purified for developed tests. 

The oxidase test and culture on TSI medium were done for isolation of *P. aeruginosa* colonies and finally 102 samples from all the isolated purified bacteria (51 human and 51 animal samples) were kept in glycerin for more studies.

The microbial sensitivity test was done based on a protocol as follows. After preparation of the standard 0.5 McFarland bacterial suspension from each of the isolated samples, a culture was prepared on the HIMEDIA-Agar (Merck, Germany). Adding different antibiotic discs to the medium, the cultures were incubated at 37 °C for 24 h and results were recorded based on the CLSI Table. Bacteria resistant to antibiotics were selected for detection of the gene’s inducing resistance using PCR method. 

The MIC test also was done using Epsilometer test (E-Test) on the HIMEDIA-Agar and the results were recorded after 24 hours at 37 °C incubation.

*DNA Extraction*

DNA was extracted from selected samples using DNA Extraction Kit (MBST-Iran) according to the manufacturer protocol. The quantitative evaluation of Extracted DNA samples were done by OD measuring with spectrophotometry. Absorption ratio of 260/280 nm was done at 1.5 to 1.9 which means the concentration of the extracted DNA was suitable for further steps of the study. The quality of DNA samples was evaluated by electrophoresis on agarose gel at 100 V (data not shown).

Specific primer pair was designed for amplifying Integron I encoding gene (Table 1).

For amplification of the target genes 10 ng of total DNA was subjected to Multiplex-PCR micro tubes in 100 microliter total volume including 10X PCR buffer, 2.5 U Taq polymerase enzyme (Cinnagen, Iran), 2 µL of each primers (20µM, Cinnagen, Iran), 2 µL of each dATP,dTTP,dGTP and dCTP (200µM Fermentase), 1.5 mM MgCl_2, _in automated Thermo cycler (MWG, *Biotech* Primus, Germany) under the following program: Denaturation step for 10 min at 95 °C, followed by 35 cycles of 30 S in 94 °C, annealing step at 55 °C for 30S and 1 min at 72 °C for elongation. 

Another Multiplex PCR reaction was done for amplifying class A ESBLs antibiotics encoding genes in *P. aeruginosa* genome based on the sequence of primer pairs shown in Table 2.

PCR done under program as mentioned above with annealing temperature at 55 °C for 30 S in 40 total cycles. Primer sets for ERIC-PCR test were also designed as Table 3 (18).

**Table 1:** Primer set sequences for amplification of Integron I encoding genes (15, 16)

**Primer names**	**Primer sequences from 5' to 3' direction**	**PCR product length**
IntI forward	GCCTTGCTGTTCTTCTACGG	
		558 bp
IntI Reverse	GATGCCTGCTTGTTCTACGG	

**Table 2:** Primer set sequences for amplification of Class A ESBLs antibiotics encoding genes in P. aeruginosa (17)

**Primer names **	**Primer sequences from 5' to 3' direction **	**Name of target gene **	**PCR product length**
VEB-1A Forward	CGACTTCCATTTCCCGATGC	blaVEB	643 bp
VEB-1B Reverse	GGACTCTGCAACAAATACGC		
GES-1AForward	ATGCGCTTCATTCACGCAC		
GES-1B Reverse	CTATTTGTCCGTGCTAAGG	blaGES	860 bp

**Table 3:** Primer set sequences for amplification of ERIC encoding genes (18).

**Primer names**	**Primer sequences from 5' to 3' direction**	**PCR product length**
ERIC forward	CACTTAGGGGTCCTCGAATGTA	
		558 bp
ERIC Reverse	AAGTAAGTGACTGGGGTGAGCG	

**Table 4.** Primer set sequences for amplification of 5 virulence factors encoding genes

**Primer names**	**Primer sequences from 5' to 3' direction**	**Name of target gene**	**PCR product length**
ExoS Forward	CGTATGAGTCAGCAAGGGCG		
		ExoS	118 bp
ExoS Reverse	GCGATGTGGTCACTGGCTTC		
ETA Forward	GACAACGCCCTCAGCATCACCAGC		
		ETA	396 bp
ETA Reverse	CGCTGGCCCATTCGCTCCAGCGCT		
oprL Forward	ATGGAAATGCTGAAATTCGGC	oprL	
			504 bp
oprL Reverse	CTTCTTCAGCTCGACGCGACG	oprL	
oprI Forward	ATGAACAACGTTCTGAAATTCTCTGCT	oprI	
			249 bp
oprI Reverse	CTTGCGGCTGGCTTTTTCCAG	oprI	
Alg Forward	TTCCCTCGCAGAGAAAACATC	Alg	
			520 bp
Alg Reverse	CCTGGTTGATCAGGTCGATCT	Alg	

**Table 5:** Primer set sequences for amplification of 4 exotoxins encoding genes in *P. aeruginosa *strains

**Primer sequences from 5' to 3' direction**	**Name of target gene**	**PCR product length**
AATCGCCGTCCAACTGCATGCG	Exo T	
		152 bp
TGTTCGCCGAGGTACTGCTC	Exo T	
CCGTTGTGGTGCCGTTGAAG	Exo U	
		134 bp
CCAGATGTTCACCGACTCGC	Exo U	
CGGATTCTATGGCAGGGAGG	Exo Y	
		289 bp
GCCCTTGATGCACTCGACCA	Exo Y	

**Table 6:** Results of isolates from human samples classification using ERIC-PCR method

**Number of strains in each group**	**Group name**
3	A
1	B
2	C
2	D
1	E
1	F
2	G
2	H
1	I
5	J
4	K
2	L
8	M
1	N
5	O
2	P
1	Q
2	R
1	S
2	T
1	U
1	Y

**Table 7:** Results of isolates from milk samples classification using ERIC-PCR method

**Number of strains in each group**	**Group name**
1	A
2	B
1	C
1	D
2	E
1	F
2	G
3	H
1	I
4	J
4	K
2	L
1	M
1	N
8	O
14	P
2	Q

**Figure 1:**
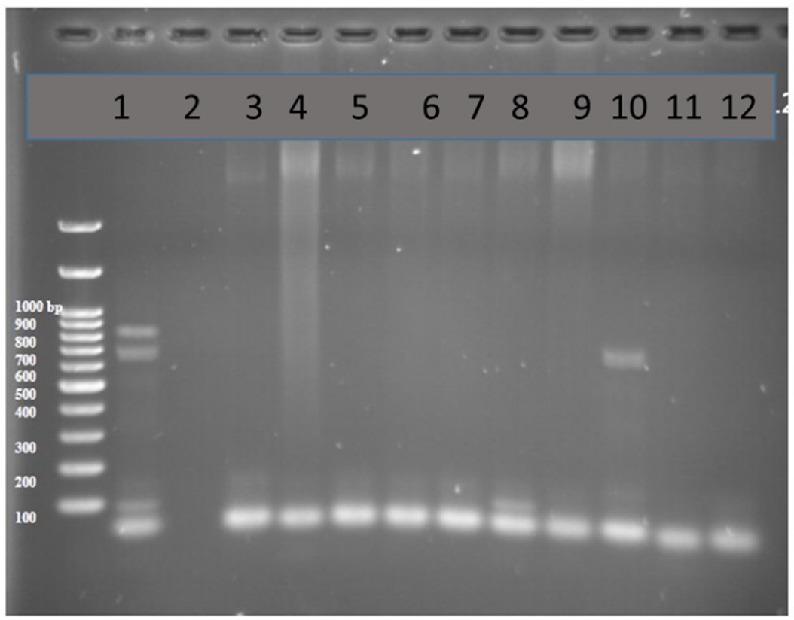
PCR products in well 1 represents two lengths of 680 bp and 643 bp as positive controls. Well number 2 represents negative control. Well number 10 contains PCR product of positive VEB encoding gene as DNA template

**Figure 2:**
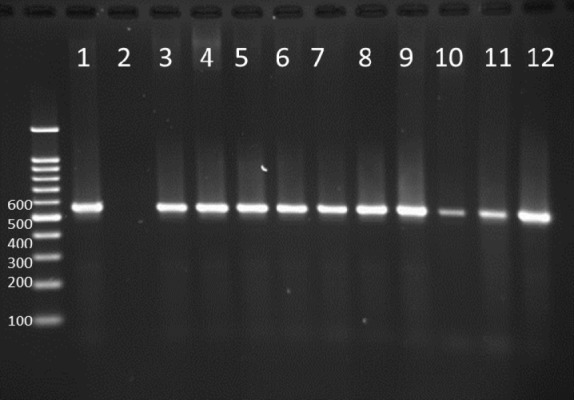
PCR products in well 1 represents positive control. Well 2 is negative control. Wells from number 3 to 12 contain PCR products of positive Integron I encoding gene as DNA template

**Figure 3:**
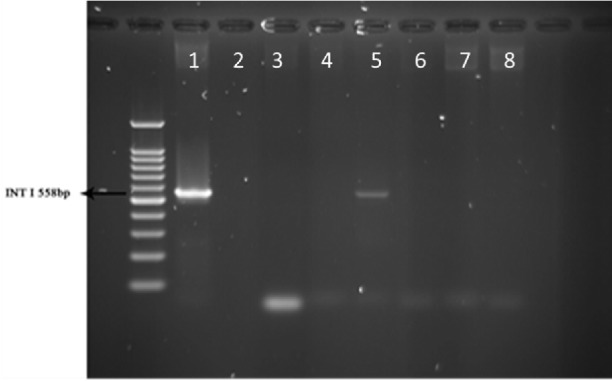
PCR products of well 1 represent positive control. The second well contains the PCR product of distilled water as template in PCR reaction as negative control. Wells from number 3 to 8 contain PCR products of animal samples amplified by INt I primers, well number 5 includes PCR product of Integron I encoding gene as DNA template

**Figure. 4:**
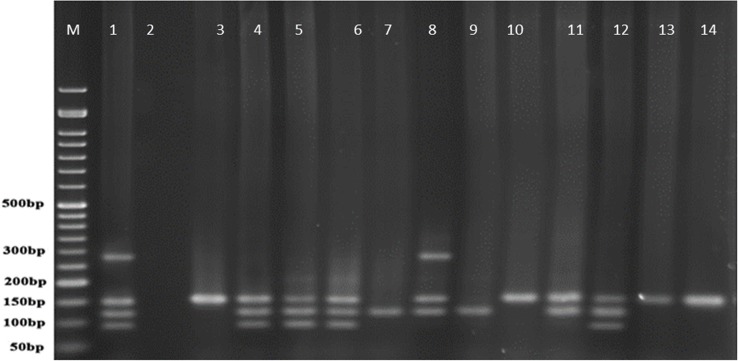
ERIC-PCR products of *P. aeruginosa *strains from human samples

**Figure 5:**
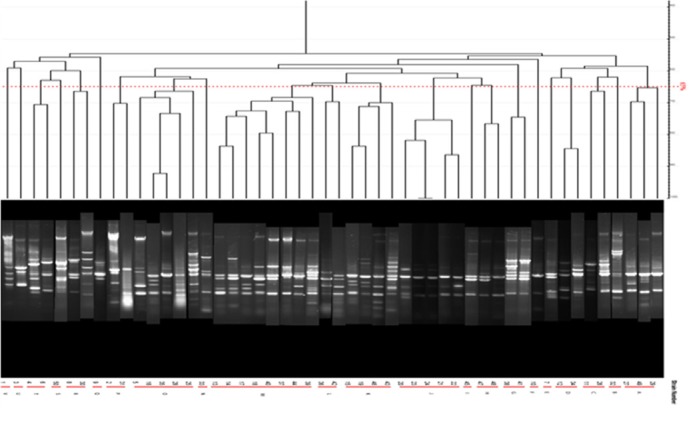
Dendrogram of the ERIC-PCR products of *P. aeruginosa *strains isolated from human samples are shown in Figure 5

**Figure 6:**
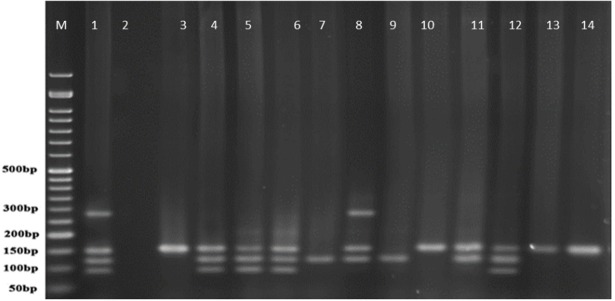
PCR products of well 1 represent positive control. The second well represents the PCR product of distilled water as template in PCR reaction as negative control. Wells from number 3 to 14 contain PCR products of human samples amplified with four specific primer pairs of exoenzymes under study

**Figure 7:**
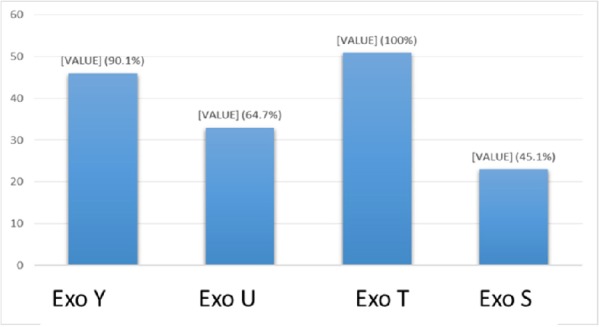
The frequency of exoenzymes Y, U, T, and S are shown in Graph 1. Exoenzyme T had the highest frequency of 100% among all studied samples taken from human patients

**Figure 8:**
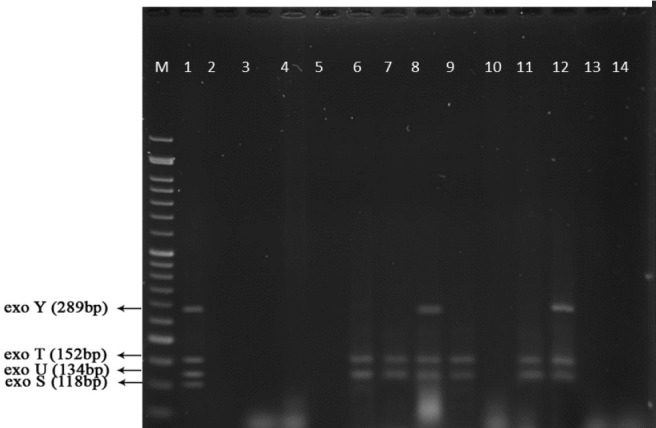
PCR products of well 1 represent positive control. The second well represents the PCR product of distilled water as template in PCR reaction as negative control. Wells from number 3 to 14 contain PCR products of human samples which are obtained from studying the exoenzymes

**Figure 9:**
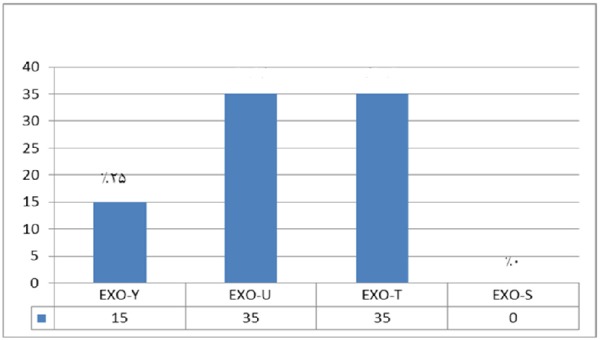
The frequency of exoenzymes Y, U, T and S are shown in Graph 1. Exoenzymes T and U had the highest frequency of 68.8% among all studied samples taken from animal isolated strains

**Figure 10:**
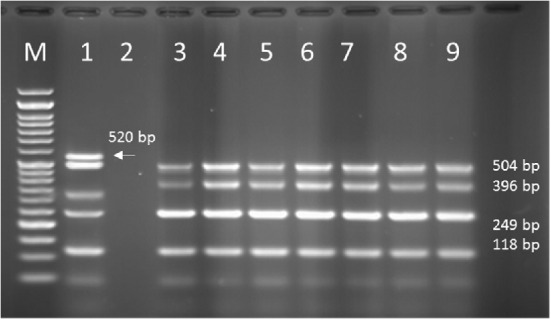
PCR products of Multiplex PCR results on DNA isolated from human on 1.5 % agarose gel electrophoresis showed specific bands of OPRL (504bp), OPRI (249 bp), ALGD (520 bp), Exo-S (118 bp), and EXT-A (396 bp) in comparison with DNA ladder.

**Figure 11:**
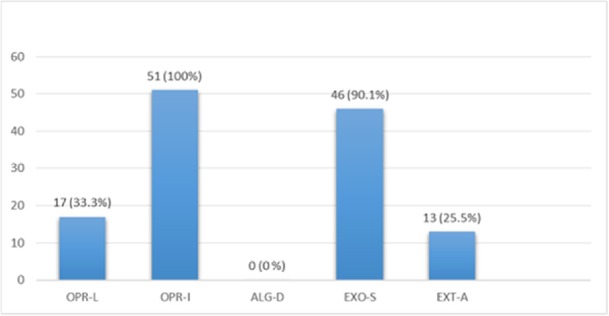
Multiplex-PCR results on strains isolated from human for detection of OPRL, OPRI, EXO-S, EXT-A, and ALGD encoding genes. ALGD and OPRI had the lowest and highest frequencies of 0% and 100% among isolated samples, respectively

**Figure 12:**
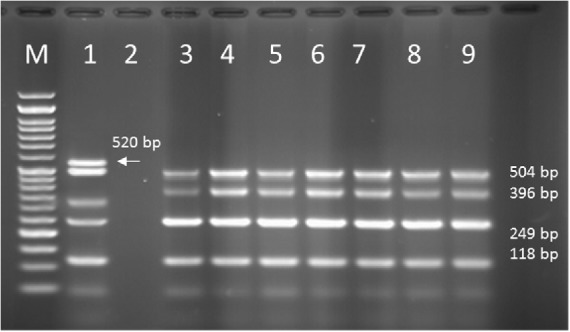
PCR products of Multiplex PCR results on DNA isolated from animal (cow) raw milk on 1.5 % agarose gel electrophoresis showing specific bands of OPRL (504 bp), OPRI (249 bp), ALGD (520 bp), Exo S (118 bp), and ext A (396 bp) in comparison with DNA ladder

**Figure 13:**
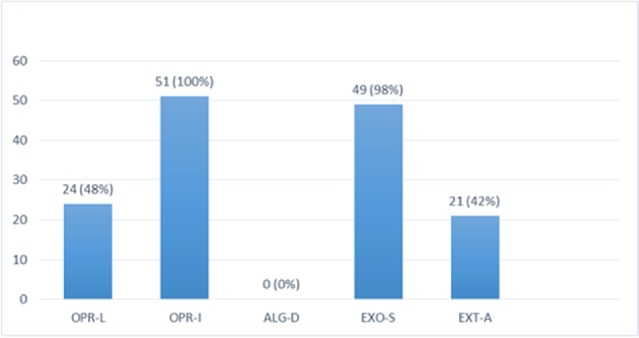
Multiplex-PCR results on strains isolated from cow raw milk for detection of OprL, OprI, exo S, ext A, and algD encoding genes; algD and OPRI have the lowest and highest frequencies of 0% and 100% among isolated samples, respectively

**Figure 14:**
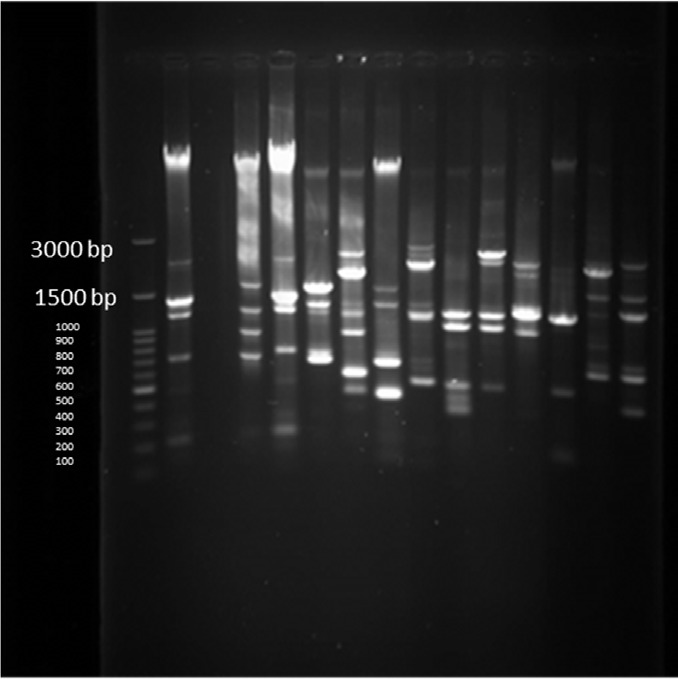
PFGE test results on 14 human samples (11 integron I positive and 1 Integron negative samples) and one Integron I positive and 1 Integron I negative samples from animals

PCR was done under the program mentioned above with annealing temperature at 52 °C for 30 S in 35 total cycles. 10 µL of all PCR products were subjected to electrophoresis on 1.5% agarose gel in TBE buffer at 100 V and were visualized under UV light by Ethidium Bromide staining. 

Five primer sets for amplification of selected five nucleotide gene sequences of virulence factors including exoenzyme S, exotxin A, oprL, oprI, and Alg were designed as Table 4 and synthesized as ordered (Takapouzist, Iran).

Three primer pairs were used for amplification of the three selected nucleotide gene sequences of eotoxin encoding genes (Table 5).

PCR was done under the program mentioned above with annealing temperature at 58 °C for 30 S in 36 total cycles and 10 µL of all PCR products were subjected to electrophoresis on 1.5% agarose gel in TBE buffer at 100 V and were visualized under UV light by Ethidium Bromide staining. Bacteria typing was done with the PFGE method. The PFGE method used in this study was based on several protocols in which after preparation of cellular suspension buffer, the plugs were prepared from the Insert gold agarose and the process of cellular lysing and plug wash were done according to the protocol. Enzymatic digestion was done using restricted endonuclease enzymes and finally all prepared plugs were subjected to electrophoresis on 1% agarose gel by CHEF-DRIII (Bio-Rad, USA) and visualized under UV light after staining with Ethidium Bromide. Results were analyzed using Gelcompar II (Applied Maths, Sint-Matens-Latem, Belgium) software and Dice analysis and were classified by Unweighted pair group method with arithmetic averages (UPGMA) (19).

For evaluation of *P*. *aeruginosa* resistant strains to antibiotics, Disc Diffusion Tests were done with commercial antibiotic discs based on CLSI standards including ciprofloxacin (5 µg), Amikacin (30 µg), Ceftriaxone (30 µg) Ceftazidime (30µg), Cefepime (10 µg), Gentamycin (10 µg), Kanamycin (30µg) Meropenem (10µg), Tetracyclin (30 µg), and Enrofloxacin (10 µg). 

E-Test (Epsilometer test) was done for evaluation of Minimum Inhibitory Concentration (MIC) on human samples using Gentamycin, ciprofloxacin, Meropenem, Imipenem, and Cefepime (Pvt.Limited-HIMEDIA, INDIANA), which was carried out with Gentamycin, ciprofloxacin, and Amikacin antibiotics.

## Results

After all samples from 51 humans and 51 animals (raw milk) were incubated at 37 °C for 24 h, the cultured colonies on Citrimid-Agar and Broth-Agar induced green-blue pigmentation while some red pigmentation was also observed (data not shown).

All isolated strains were Catalase, Citrate, Methyl-Red, Urease and Oxidase positive and Indole, VP (Voges Proskauer test) negative as expected.

*Multiplex-PCR results on strains isolated from human for detection of VEB and GES Beta-lactamase genes*

Multiplex-PCR was carried out for detection of Beta-lactamase VEB and GES encoding genes in sample strains isolated from human. Results showed that 2% of the total strains taken from 51 human samples were VEB positive and none of the 51 human samples were amplified with GES specific primers (Figure 1).

*PCR results on strains isolated from animals for detection of VEB and GES Beta-lactamase genes*

The same Multiplex-PCR test was done on 51 strains of raw milk and results were negative for VEB and GES specific primers and none of the strains from animals contained Beta-lactamase VEB and GES encoding genes, respectively (data not shown).

*PCR results on strains isolated from human for detection of Integron I encoding gene*

PCR test done on 51 strains of the ones isolated from humans for detection of Integron I encoding gene with Int-1 primers led to detection of PCR product with length of 558 bp (as expected) in 11 out of 51 samples (21.5%, Figure 2).

*Multiplex-PCR results on strains isolated from animals for detection of Integron I encoding gene*

PCR test done on 51 strains of the ones isolated from animals for detection of Integron I encoding gene with Int-1 primers showed that the Integron I had a lower frequency than in human with t ratio of 1 out of 51 samples (1.96 %, Figure 3).

*Results of ERIC-PCR on isolates taken from human samples*

Dendrogram of ERIC-PCR method on human isolated samples revealed 22 different groups in 1500 bp, 1000bp, 500bp, and 100 bp regions with the cut off value 65%. Most of the samples belonged to M group (8 isolates) which contained 16% of all human studied samples showing the maximum similarities (Table 6).

ERIC-PCR results on isolates from cow milk samples revealed 17 graphs of 1500 bp, 1000bp, 500bp, and 100 bp with 68% cut off with the highest frequency of 28% (14 strains) with the high similarities among P group (Table 7).

ERIC-PCR products and Dendrogram of *P. aeruginosa* strains from milk samples are not shown.

*Multiplex-PCR results on strains isolated from human for detecting exoenzymes T, Y, U, and S encoding genes*

Multiplex-PCR results for detection of four exoenzymes Y, U, T, and S encoding genes with PCR product of 289 bp, 134 bp, 152 bp, and 118 bp in length, respectively showed that exoenzyme T had a higher frequency in the strains under study with the value of 100% among them (Figure 6). Frequencies are shown in Figure 7.

*Multiplex-PCR results on strains isolated from animals for detection of exoenzymes T, Y, U, and S encoding genes*

In comparison with human isolated strains, strains isolated from animal samples showed a lower frequency of exoenzymes encoding genes, as shown in Figure 5. PCR on exoenzyme S was negative while the frequency of exoenzyme Y in 15 isolates was 25% and the record for exoenzymes U and T was 68.8% (Figure 8).

Figure 9 represents Multiplex-PCR results for detection of four exoenzymes Y, U, T, and S encoding genes with PCR product of 289 bp, 134 bp, 152 bp, and 118 bp in length, respectively in strains from animal samples.

*Multiplex-PCR results on strains isolated from human for detection of OPRL, OPRI, EXO-S, EXT-A, and ALGD encoding genes*

Multiplex PCR results on DNA isolated from human samples with specific primer pairs showed that OPRI had the highest frequency of 100% among these five mentioned genes. PCR product electrophoresis on 1.5% agarose gel showed the specific bands of OPRL (504bp), OPRI (249 bp), ALGD (520 bp), Exo-S (118 bp), and EXT-A (396 bp) as shown in Figure 10. The frequency of OPRL, OPRI, EXO-S, EXT-A, and ALGD encoding genes are shown in Figure 11.

*Multiplex-PCR results on strains isolated from animals (cow raw milk) for detection of OPRL, OPRI, EXO-S, EXT-A, and ALGD encoding genes*

Multiplex PCR results on DNA isolated from milk samples with specific primer pairs showed that OPRI had the highest frequency of 100% among the five genes under study. PCR product electrophoresis is shown in Figure 12 the frequencies of OPRL, OPRI, EXO-S, EXT-A, and ALGD encoding genes are shown in Figure 13.

Exotoxin A encoding gene detected in more than 90% of known *P*. *aeroginosa *strains, coding the main venom of the bacteria, causes a high rate of mortality in infected patients (20). In this study 13 out of 51 isolates from human samples (25.5%) were positive which is relatively low in comparison with the studies reported by other researchers. This finding also proves the high frequency of Exotoxin A encoding gene present in *P*. *aeroginosa *isolates. 

*PFGE Results*

PFGE test carried out on 14 human samples (11 integron I positive detected and 1 Integron negative samples) and one Integron I positive and 1 Integron I negative samples selected among isolates of animal cow milk samples because of the large number of samples. Figure 14 shows that all human and animal integron I negative tested samples were negative in PFGE results but all Integron I positive samples showed different products in different lengths which were diagrammed using dendrogram software (Figure 14).

*Disc Diffusion Test Results*

The microbial sensitivity test was done on 51 isolates of human samples showing the highest resistance to Ampicillin and Cefepime (100%) and the lowest resistance to Amikacin (80.3%) according to the protocol mentioned in material and methods section. The most frequent resistance to more than one antibiotic in one isolate was also observed in 16 isolates with the high number of resistance against 3 antibiotics. MDR was also seen among isolates from cow milks. The most prevalent resistance to 3 antibiotics (Kanamycin, Ampicillin, and Tetracyclin) was observed in 25 isolates, none of them showing resistance against all studied antibiotics.

*E-Test results for evaluating resistance to antibiotics in the human isolated studied strains*

E-Test was carried out for evaluating the resistance of human isolated strains to Meropenem, Imipenem, Cefixim, Amikacin, Gentamycin, and Cefepim, according to the standard protocol of CLSI in comparison to standard strain as positive control. The minimum and maximum effective concentrations were 0.1 µg and 8 µg of Amikacin and Imipenem in human isolated samples, respectively. All of the studied isolates were resistant to Meropenem (Data not shown).

*E-Test results for evaluating of resistance to antibiotics in the animal isolated studied strains*

The same test was done on animal (cow milk) strains resistance against 3 antibiotics of Amikacin, Gentamycin, and Ciprofloxacin. The minimum and maximum effective concentration were 0.125µg and 8 µg of Ciprofloxacin and Amikacin and in animal isolated samples (Data not shown).

## Discussion

Many studies have been carried out for detecting the mechanisms in which *P*.*aeruginosa *becomes MDR pathogen. Genes encoding several enzymes that cause resistance to Beta-lactam antibiotics are classified in Class A such as VEB and GES enzyme encoding genes. In this study Multiplex-PCR on human isolated strains revealed the frequency of VEB and GES encoding genes as 3.9% and 0, respectively. None of the two target genes were detected in the studied animal strains by Multiplex-PCR method. Bokaeian and his colleagues detected 6.89% of total 116 samples as ESBLs positive and disclosed the frequency of 16.3% MDR *P.aeruginosa* strains isolated from patients in Zahedan-Iran; they also recorded the Ciprofloxacin and Piperacillin as the most efficient anti-*Pseudomonal* agents (21). It can be realized that resistance to antibiotics in isolates studied in our study comes from the presence of other probable resistance factors. The microbial sensitivity test was done on 51 isolates of human samples showing the highest resistance to Ampicillin and Cefepime (100%) and the lowest resistance to Amikacin (80.3%) according to the protocol mentioned in material and methods section. The most frequent resistance to more than one antibiotic in one isolate was also seen in 16 isolates with the high number of resistance against 3 antibiotics in human isolates. MDR was also seen among isolates of cow milks. The most prevalent resistance to 3 antibiotics (Kanamycin, Ampicillin, and Tetracycline) was observed in 25 isolates, none of them being resistant to all studied antibiotics. E-Test results showed resistance of human isolated strains to Meropenem, The same test was carried out on animal (cow milk) strains against 3 antibiotics of Amikacin, Gentamycin, and Ciprofloxacin with the minimum and maximum effective concentrations of 0.125 µg and 8 µg of Ciprofloxacin and Amikacin, respectively showed the highest rates of drug resistance to Ampicillin (100%) compared to the antibiotics of Imipenem, Ciprofloxacin, Enrofloxacin, Gentamicin, and Amikacin. Franco showed that 100% of all samples taken from hospitalized patients in Brazil were resistant to Imipenem (22). Doosti *et al.* in 2014 reported the frequency of 55.1% resistance to Imipenem among strains isolated from clinical patients in Iran (23). Integrons are motile genetics elements which transfer important sequences between bacteria which lead to MDR pathogen formation in Gram-negative species (12). Horizontal transfer of resistance encoding genes with Integrons caused increasing in *P*. *aeroginosa* resistant strains (9). Integrons class I are the most common integrons found in clinical isolated *P*. *aeroginosa* strains. Prevalence of Integron I encoding gene was 1.96% in our study that was much lower than the other studies worldwide and this could be because of the difference in hygienic protocols among countries and various geographic regions in Iran. Precise typing of *P*. *aeroginosa *different strains is needed for control and epidemiological monitoring of the bacteria spreading and detecting new strains in a known bacterial population. Among different molecular-based methods for achieving this aim, ERIC-PCR is one of the valuable rapid typing methods that can be used easily in every molecular biological equipped labor (13). ERIC-PCR test done in this study showed 65% and 68% similarity in isolates taken from 51 animal and 51 human samples, respectively. Dendrogram drew based on the ERIC-PCR results revealed that bacterial samples especially from animals (cow milk) had a common genetic progenitor.

Syrmis in 2004 used ERIC-PCR for typing the *P. aeruginosa* strains and results showed 6 main groups of 58 isolates out of 163 total studied isolates (24). Dendrogram of our study also showed the least similarity among clusters. Most of the isolates were included in the cluster M and P among human and animal taken samples, respectively. In this study PFGE was done on 14 samples (11 human integrin I positive and one Integron I negative, one animal Integron I positive and one negative sample). Results showed the most similar patterns in samples taken from different origins (A5, A6) that seems a deviation from the same species. The origin of samples from human patients in this study was urine samples (A-1, C-7, A-4, H-32, C-15, H-22, H15-13 and A-2), lesion (A-6), and trachea (A-5 and A-15). Urine samples were shown to be the most positive Integron I which had less similarity. Prevalence of exoenzyme S in samples isolated from animals (cow milk) was negative and the frequency of Exo Y, Exo T, and Exo U were 25%, 68.6%, and 68.6%, respectively. 

Alginate is another virulence factors in *P*. *aeroginosa *bacteria which is encoded by a group of related genes including algD. The prevalence of algD in our study was 0 and this may be because of the source of isolates. OprL is one of the lipoproteins of virulence factors in the bacteria that is also a marker for detection of *P*. *aeroginosa *species. De vos first in 1997 studied the presence of OprL encoding gene by multiplex PCR on 20 different *Pseudomonas *species (25), the results showed that *P*. *aeroginosa *is the only species containing OprL gene with the sensitivity and specificity of 100% and 74% by PCR method. In our study, the prevalence of these factors was recorded as OprI (100%) and OprL (33.3%) in 51 total isolates taken from human samples and OprL (48%) and OprI (100%) in a total of 51 samples of isolates from cow raw milk, respectively.

Render in 1996 suggested that the size and count of PCR products of RAPD-PCR method in comparison to PFGE for genotyping the *P.aeruginosa* strains are highly dependent to designed primers; they also found 8-15 different PCR product bands in the range of 100-1500 bps. They further revealed the same pattern of results in homogenous strains using both methods. Therefore, PFGE could be considered as golden standard to confirm the RAPD-PCR results (19). E-test results of our study also showed the difference between strains of animal studies in comparison to human results which can be another proof of genetic diversity occurred in strains of the same geographical origin but isolated from two different hosts of human and animal. Exotoxin A encoding gene was detected in more than 90% of known *P*. *aeroginosa *strains, coding the main venom of the bacteria and causing the high rate of mortality in infected patients (20). In this study 13 out of 51 isolates from human samples (25.5%) were positive showing a lower rate in comparison with the findings of similar studies. This finding also confirms the high frequency of exotoxin A encoding gene in *P*. *aeroginosa *isolates. Amini and colleagues showed that 90% of the total isolates in their study encoded ETA gene (26) which was higher than 25.5% of human samples and 42% of animal samples detected in our study. 

## Conclusion

In general, the present study showed the high value of genetic diversity among isolates from animal and human samples with different progenitors, but the clones classified in one cluster revealed the same source of infection. The differences between these findings and those of other researchers could be attributed to different sources of human sampling (lesion, urine, etc.), the environmental conditions, and the sensitivity of various hosts.
